# An objective and subjective health literacy analysis among heart transplant recipients

**DOI:** 10.3389/fpubh.2025.1575256

**Published:** 2025-12-08

**Authors:** Lili Diána Hajnes-Szabó, Edit Czeglédi, János Pilling, Balázs Sax, Béla Merkely, Alexandra Assabiny

**Affiliations:** 1 Department of Internal Medicine and Haematology, Semmelweis University, Budapest, Hungary; 2 Institute of Behavioural Sciences, Semmelweis University, Budapest, Hungary; 3 Heart and Vascular Centre, Semmelweis University, Budapest, Hungary

**Keywords:** health literacy, objective health literacy, subjective health literacy, quality of life, heart transplant recipients

## Abstract

**Background:**

Health literacy (HL) is associated with patient adherence, healthcare utilization, patient self-management, however there is limited data available on how it should be interpreted and measured among heart transplant recipients.

**Methods:**

In a cross-sectional study among heart transplant recipients (*n* = 98) under follow-up at Semmelweis University Heart and Vascular Centre, HLS-EU-Q47 and Newest Vital Sign test were used to measure objective and subjective HL.

**Results:**

The HLS-EU-Q47, a measure for subjective HL, showed that 49.5% of heart transplant recipients had excellent, 35.1% sufficient, 14.4% problematic and 1% inadequate HL. For objective HL, measured with the NVS test, the frequency of HL categories (adequate HL 49%, possibility of limited HL 26.5%, high likelihood of limited HL 24.5%) was significantly different. We were not able to identify a significant predictor of subjective HL. However, objective HL showed a significant association with both age (*β* = −0.445, *p* < 0.001) and educational attainment (*β* = 0.212, *p* = 0.023). There was no significant association between HL and health risks or health-promoting behaviors (alcohol consumption, physical exercise).

**Conclusion:**

The results of our research indicate that subjective and objective (performance-based) HL are two different concepts and should be treated separately. Finding ways to improve HL among heart transplant recipients should be a priority and requires a complex assessment process, a multi-faceted approach both for caregivers and stakeholders.

## 1 Introduction

In recent years, health literacy (HL) has become a central concept in medicine. From 2000 to 2025 October, the Web of Science database identified more than 17,000 scientific publications on this topic. The World Health Organization defines health literacy as “representing the personal knowledge and competencies that accumulate through daily activities, social interactions and across generations. Personal knowledge and competencies are mediated by the organizational structures and availability of resources that enable people to access, understand, appraise, and use information and services in ways that promote and maintain good health and well-being for themselves and those around them” (1). The European Health Literacy Survey (HLS-EU), was conducted in eight European countries and the pooled prevalence of low health literacy in European Union member states ranges from 27 to 48% (2). A study was launched in Hungary using the same measures, which found that more than half of the adults (52%) had limited HL (3).

People with limited HL lead unhealthier lifestyles, they are less likely to attend disease screening, and to be involved in health promotion programs, and have poor adherence when ill. Limited HL also means less ability to self-manage chronic conditions, and often results in more hospitalizations, poor quality of life (QoL), higher mortality rates, healthcare costs and higher rates of premature death compared to people with adequate HL (2, 4).

HL is particularly important among patients with chronic conditions, as it is an important determinant of their health status and the effectiveness of treatment and for that reason, a growing number of methods are being used to improve HL of patients with chronic diseases (4–7).

The importance of HL among organ transplant recipients is increased by the fact that patients need to comply with complex treatment protocols before and after surgery, and therefore patient cooperation is essential during this costly procedure and planned interventions are needed to address the topic of low health literacy in this patient group (8). According to a prospective, multi-center cohort study, which measured nursing complexity and health literacy as determinants of patient outcomes, both high nursing complexity and inadequate HL independently and jointly play a role in adverse patient outcomes. Given the complexity of the posttransplant care, this aspect is increasingly important for heart transplant patients and highlights the fact that finding ways to measure and interpret health literacy is crucial (9).

With regard to the organ transplant population, most research on HL has been conducted among kidney and liver transplant recipients. People with limited HL are less likely to be put on the kidney or liver transplant list or, if they are, they would be more likely to be removed from it (10, 11). Among both liver and kidney transplant recipients, limited HL has been associated with poor adherence to immunosuppressive and other medications (12, 13). Among kidney transplant recipients, several studies have shown that limited HL has been associated with a higher risk of graft rejection and mortality (10, 13).

There has been very limited research on HL among heart and lung transplant recipients, with just a few publications on this topic (14, 15). In a Swedish study, low or marginal HL was found in 21% of lung transplant patients, while 79% of patients had adequate HL (15).

The BRIGHT study was conducted among 1,365 heart transplant recipients from 11 countries on 4 continents and the results show that 33.1% of participants had poor HL. The strength of the BRIGHT study is the high sample size. However, a major limitation of the study is that HL was assessed using a single, subjective question (14). It was suggested that further research with a more thorough methodology is needed (16).

We investigated the associations of health literacy (HL) with quality of life (QoL) and health behaviors among heart transplant recipients. We hypothesized that higher HL would be associated with higher educational attainment, better subjective QoL, lower rates of smoking and alcohol use, and increased physical activity.

## 2 Materials and methods

The study followed a cross-sectional, questionnaire-based observational design and was carried out at the Semmelweis University Heart and Vascular Centre (Budapest, Hungary). This study complies with the Declaration of Helsinki and Declaration of Istanbul. It was approved by Semmelweis University Regional and Institutional Committee of Science and Research Ethics (SE RKEB number: 174/2016).

### 2.1 Participants and procedure

We included adult heart transplant recipients who were in long-term follow-up at the Semmelweis University Heart and Vascular Centre. Participants were eligible for inclusion if they met the following criteria: we included all heart transplant recipients who were treated at Heart and Vascular Centre after heart transplant after the post-transplant consultation. Exclusion criteria were as follows: heart transplant recipients who did not attend a follow up during the data collection period (October 2016–October 2017). Heart transplant recipients who have not received the post-transplant consultation from doctors. Heart transplant recipients who had serious health issues which prevented them from being able to be interviewed.

Written informed consent was obtained from all patients, respondents were free to leave at any time. Data were gathered in structured, face-to-face interviews between October 2016 and October 2017. The interviews took place at the treatment facility, either in the outpatient department or the post-operative transplant ward with a participant and the interviewer in attendance, each lasted approximately 50–80 min. The same interviewer (first author) conducted each paper-and-pencil interview. Altogether 100 patients were involved in the survey, data from 98 participants were finally used due to missing responses. One hundred and twenty-seven heart transplant recipients were treated at Semmelweis University during the period in question.

At Semmelweis University Heart and Vascular Centre all post-heart transplant patients have the opportunity to participate in a post-transplant consultation which includes the treating doctors, recipients, and caregivers as well. Patients can ask their questions about the surgery and aftercare during the session. Heart transplant recipients also receive a written information leaflet prepared by the doctors before the consultation, which contains key information on life after heart transplantation (HTx).

### 2.2 Measures

We recorded major sociodemographic characteristics of respondents (gender, age, levels of education, marital status) and collected information on their possible medical professional background and the date of the transplant (21).

Two different instruments were used to measure HL. Firstly, the Hungarian version of the validated Health Literacy Survey Questionnaire (HLS-EU-Q47) (3, 17, 18).

Responses were rated on a four-point Likert scale (1 = very difficult, 2 = difficult, 3 = easy, and 4 = very easy). The total score represents a general HL index which consists of three sub-indices, such as health care (“It measures the ability to access, understand and interpret medical information and make health related decisions”) disease prevention (“It evaluates accessing, understanding and interpreting information on risk factors and the ability to judge the relevance of this information”), and health promotion (“It measures the ability to access, understand and interpret health promotion related information and form an opinion accordingly”) (18). Higher scores reflect higher levels of HL, and four categories of health literacy can be distinguished: inadequate (0–25 points), problematic (>25–33 points), sufficient (>33–42 points) and excellent (>42–50 points) (18). The internal consistency of the entire questionnaire (Cronbach’s alpha = 0.94) and its sub-indices (Cronbach’s alpha = 0.85–0.88) proved to be acceptable in the present sample.

The validated Hungarian version of the Newest Vital Sign (NVS) test was the other instrument of HL (3, 19, 20). It contains six questions about an imaginary ice cream nutrition label where basic mathematical, reading and text comprehension skills are required to solve the questions. This measure defines three levels of functional HL according to the number of correct answers (from 0 to 6 points): high likelihood of limited HL (0–1 point), possibility of limited HL (2–3 points) and adequate HL (4–6 points) (20). The internal consistency of the test was acceptable in the present sample (Cronbach’s alpha = 0.82).

Quality of life was assessed with two questions, which were used for our study purposes, and are not validated measures: 1. How would you rate your current quality of life on a scale from 1 to 5, where 1 is the worst and 5 is the best? 2. How would you rate your pre-transplant quality of life on a scale from 1 to 5, with 1 being the worst and 5 being the best?

Regarding *health risks behaviors*, we asked questions about smoking and alcohol consumption. These questions are part of the validated and widely used HLS-EU-Q86 measure (21). Do you smoke or have you ever smoked (cigarettes, cigars, pipes)? Did you drink any alcoholic beverages (beer, wine, spirits, cider or other) in the last 30 days? How many times have you consumed alcohol in the last 30 days? On days when you drink alcohol, how much do you usually drink?

With regard to *health-promoting behaviors*, we gathered information about physical activity. These questions are part of the validated and widely used HLS-EU-Q86 measure (21). During the past month, how often did you exercise for at least 30 min, e.g., running, walking, cycling?

### 2.3 Statistical analyses

To estimate the internal reliability of the scales, we calculated Cronbach’s alpha coefficients. Intraclass Correlation Coefficient (ICC) analysis was used to assess the level of agreement between the two continuous measurement tools of health literacy. A two-way mixed-effects model with absolute agreement was applied (22). To compare HL of heart transplant recipients with international reference values, we used a chi-square test. Potential predictors of HL were tested by multiple linear regression analysis, using the enter method. A *post hoc* power analysis was conducted using G*Power (version 3.1.9.7) to assess the achieved statistical power for the multiple linear regression analysis (23). The levels of the quality of life before and after surgery were assessed with a paired sample *t*-test. As effect size, Cohen’s *d* was calculated. Group comparisons on continuous variables were conducted using independent-samples *t*-tests. Linear relationships were tested with Spearman’s rank correlation analysis. Analyses were performed with SPSS 21.0.

## 3 Results

The majority of the sample (*n* = 98) was male (84%), with a mean age of 51.6 (SD = 10.48, range: 23–71) years. On average, 28.5 (SD = 41.2, range: 0.9–192.4) months passed between heart transplantation and data collection. Other demographic data are presented in Table 1.

**Table 1:** Demographic characteristics of the sample.

Variables	Categories	*n* (%)
Gender	Males	82 (83.7)
	Females	16 (16.3)
Marital status	Single	17 (17.3)
	Married	59 (60.2)
	Not living together/Divorced	17 (17.3)
	Widowed	5 (5.1)
Education	Less than high school	56 (57.1)
	High school graduate	23 (23.5)
	College graduate or more	19 (19.4)
Healthcare professional background	Yes	9 (9.2)	No	89 (90.8)

### 3.1 Health literacy

The Intraclass Correlation Coefficient (ICC) calculated between the two measurement instruments—namely HLS-EU-Q47 and NVS test—yielded negative values (single measures: –0.004 [95% CI: −0.012, 0.011]; average measures: –0.007 [95% CI: −0.025, 0.022]), indicating a lack of agreement and potentially systematic disagreement between the tools. According to established guidelines, ICC values below 0.50 reflect poor agreement, with negative values suggesting that the variability due to measurement error exceeds the variability between subjects (22). These findings imply that the two instruments should not be considered interchangeable for assessing health literacy. The observed discrepancies may be attributable to differences in their conceptual foundations or scaling methodologies. Consequently, subsequent analyses were conducted separately for each instrument.

The mean score for the HLS-EU-Q47 and its sub-indices was above 40.0 points in all cases. In comparison with representative Hungarian data and representative data from HLS-EU, we found that excellent HL was significantly more frequent among heart transplant recipients, while problematic and inadequate HL were less frequent (Table 2) (3, 21).

**Table 2:** Percentage frequency distribution of health literacy categories (HLS-EU-Q47) and comparison with reference data.

Variables	Sample	Inadequate	Problematic	Sufficient	Excellent	*χ^2^*(df) *p*
General health literacy index	Present	1.0	14.4	35.1	49.5	–
	Reference 1	19	33	38	10	*χ^2^*(3) = 178.065 *p* < 0.001
	Reference 2	12.4	35.2	36.0	16.5	*χ^2^*(3) = 86.066 *p* < 0.001
Health care literacy index	Present	0.0	13.4	38.1	48.5	–
	Reference 1	18	27	40	15	*χ^2^*(3) = 96.558 *p* < 0.001
	Reference 2	12.1	28.8	39.1	19.9	*χ^2^*(3) = 59.427 *p* < 0.001
Disease prevention literacy index	Present	1.0	14.4	30.9	53.6	–
	Reference 1	21	30	36	13	*χ^2^*(3) = 149.991 *p* < 0.001
	Reference 2	13.7	29.1	35.9	23.1	*χ^2^*(3) = 66.739 *p* < 0.001
Health promotion literacy index	Present	3.1	16.7	36.5	43.8	–
	Reference 1	25	29	35	11	*χ^2^*(3) = 117.074 *p* < 0.001
	Reference 2	30.8	20.1	49.1		*χ^2^*(2) = 43.356 *p* < 0.001

Reference 1: representative Hungarian data (3), Reference 2: representative data for 8 European countries (Hungary is not included) (21).

However, for the NVS test, the frequency of HL categories did not differ significantly from the European representative data, but in comparison with Hungarian representative data, HL of heart transplant recipients had significantly worse results (Table 3) (19, 21).

**Table 3:** Percentage frequency distribution of health literacy categories (NVS) and comparison with reference data.

Sample	High likelihood of limited literacy	Possibility of limited literacy	Adequate literacy	*χ^2^*(df) *p*
Present	24.5	26.5	49.0	–
Reference 3	12	20	69	*χ^2^*(2) = 20.718 *p* < 0.001
Reference 2	21.2	23.5	55.3	*χ*^2^(2) = 1.591 *p* = 0.451

Reference 2: representative data for 8 European countries (Hungary is not included) (21). Reference 3: representative Hungarian data (19).

### 3.2 Predictors of health literacy

Potential predictors of HL were tested using multiple linear regression analysis. For the first model, the General HL Index of the HLS-EU-Q47 was the dependent variable, while for the second model, the NVS test was the dependent variable. The independent variables were gender, age, education, and time since HTx. On the basis of our results, we could not identify any significant predictor for the general HL index (HLS-EU-Q47) and the variance explained by the model was also zero (Model 1, Table 4). However, HL measured with the NVS test showed a significant association with both age and education. HL decreased with increasing age (*β* = −0.445, *p* < 0.001), and was higher among those with at least high school graduation than among those with lower education (*β* = 0.212, *p* = 0.023). The variance explained by the model was 21.2% (Model 2, Table 4). Due to the limited sample size (*n* = 98), a *post hoc* power analysis was conducted using G*Power (version 3.1.9.7) to assess the achieved statistical power for the multiple linear regression analysis (Model 2) (23). Given an observed effect size of *f*^2^ = 0.269 (calculated from the second model’s coefficient of determination [*R*^2^], using Cohen’s formula *f*^2^ *= R*^2^*/*(*1 − R*^2^)), an alpha level of 0.05, a total sample size of 98 participants, and 4 predictors, the analysis revealed a statistical power of approximately 0.99 (24). This indicates that the study had a very high probability of detecting a true effect of this magnitude.^1^

**Table 4:** Predictors of health literacy.

Independent variables	Model 1 General health literacy index (HLS-EU-Q47)			Model 2 Newest Vital Sign test		
	*β*	*t*	*p*	*β*	*t*	*p*
Constant		9.963	<0.001		6.013	<0.001
Gender (1. male, 2. female)	−0.030	−0.293	0.770	0.036	0.389	0.698
Age	0.181	1.692	0.094	−0.445	−4.730	<0.001
Levels of education (1. less than high school, 2. at least high school graduate)	−0.070	−0.676	0.501	0.212	2.318	0.023
Time since heart transplant surgery (month)	0.003	0.032	0.975	0.018	0.195	0.846
Adjusted *R*^2^	0.0%			21.2%		

### 3.3 Quality of life

**Figure 1:**
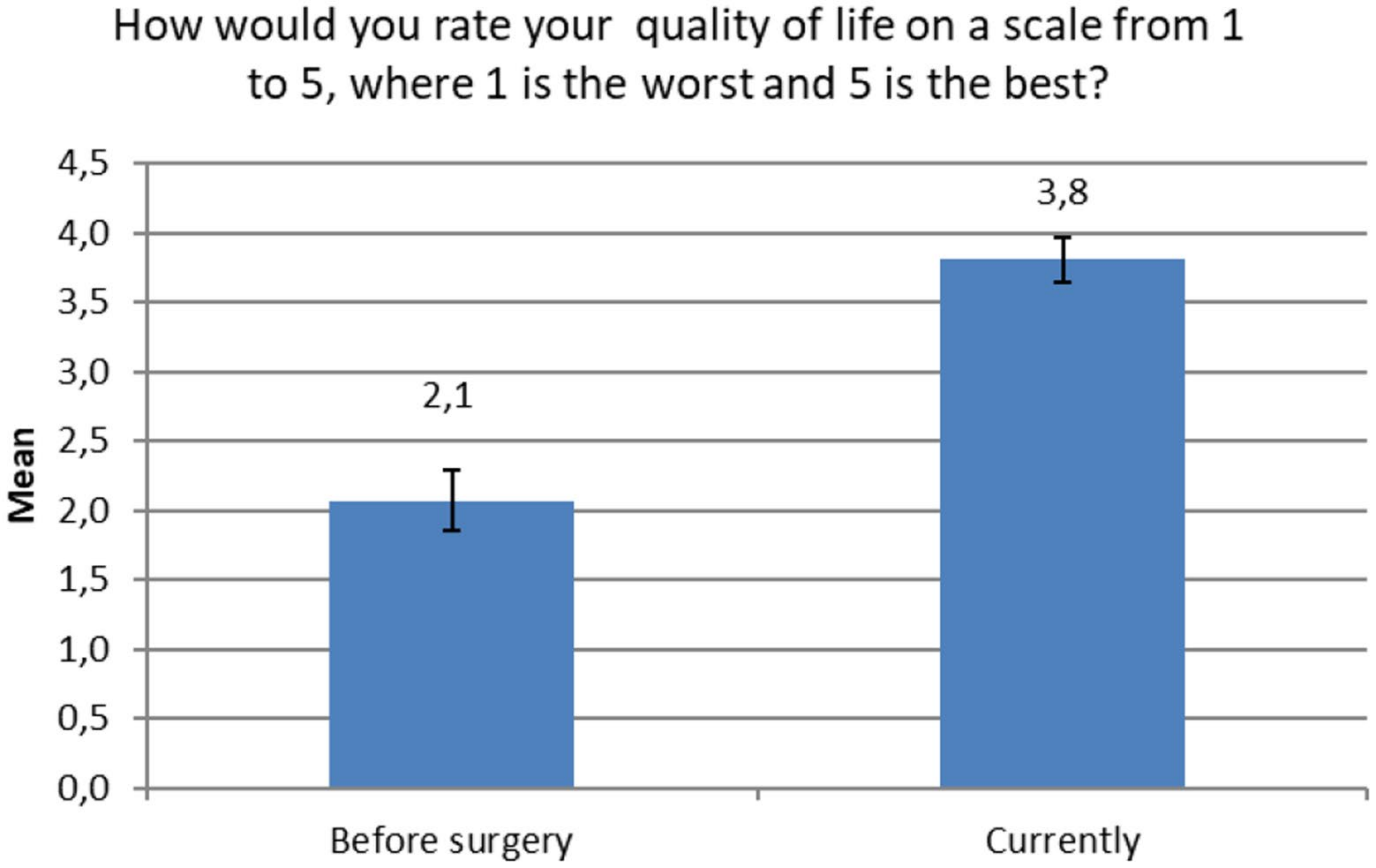
Changes in perceived quality of life. The figure shows the 95% confidence intervals of the means.

The results of the paired sample *t*-test showed that the current perceived QoL of the participants was significantly and largely better than before HTx (*t*(97) = −12.171, *p* < 0.001, Cohen’s *d* = 1.76, Figure 1).

The results of the correlation analysis showed that the current QoL had a significant, weak, positive association with the HLS-EU-Q47 questionnaire scores, both in the case of the general HL index and the sub-indices. However, HL operationalized with the NVS test was not significantly associated with subjective QoL (Table 5).

**Table 5:** The relationship between health literacy and quality of life.

Variables	Current quality of life
General health literacy index	0.24*
Health care literacy index	0.21*
Disease prevention literacy index	0.21*
Health promotion literacy index	0.24*
Newest Vital Sign test	0.05

Spearman’s rank correlation coefficients, *p < 0.05.

### 3.4 Health risks and health-promoting behaviors

With regard to smoking, 2.0% of respondents (*n* = 2) were smokers at the time of the data collection, 73.5% (*n* = 72) had been smokers in the past but had already quit, while 24.5% (*n* = 24) had never smoked.

A quarter of the respondents (25.5%, *n* = 25) reported having consumed alcohol in the past 30 days. Daily alcohol consumption affected 3.1% (*n* = 3). In this sample of heart transplant recipients, 1.0% (*n* = 1) consumed alcohol 4–5 times a week, 4.1% (*n* = 4) 2–3 times a week, 3.1% (*n* = 3) once a week, while 7 respondents each (7.1–7.1%) reported drinking alcohol 2–3 times a month or only once in this period. In terms of the amount of alcohol consumed on one occasion in the past month, we found that 2.0% of the respondents (*n* = 2) had 3–4 drinks, 17.3% (*n* = 17) had 1–2 drinks, 6.1% (*n* = 6) had less than one drink occasionally.

Finally, our results showed that half of the participants (50%, *n* = 49) had exercised (e.g., running, walking, and cycling) for at least 30 min almost every day in the last 30 days. A fifth of them (21.4%, *n* = 21) had done so a few times a week, 8.2% (*n* = 8) a few times in the past month. A further fifth of the sample (20.4%, *n* = 20) did not exercise at all.

Overall, no significant or even marginally significant relationships were found with any of the measured aspects of HL for alcohol consumption, and physical exercise. The authors will provide the data upon request.

## 4 Discussion

This cross-sectional study is the first one which explores the health literacy of heart transplant recipients with two different questionnaires simultaneously to our knowledge. We found very different HL results depending on the measurement tool used. The frequency of limited HL (inadequate and problematic) was 15.4% measured with the HLS-EU-Q47, however, measured with the NVS test, 24.5%. HLS-EU-Q47 measures the self-perceived level of health literacy by evaluating the ability to access, understand, appraise and apply health information to make decisions regarding health, while, NVS is an objective screening tool, which evaluates mathematical, reading and text comprehension skills (18, 20). Comparing HLS-EU-Q47 results with national data and representative data from HLS-EU, we found that our sample showed better results in all aspects for subjective HL (3, 21). This may be explained by the knowledge and experience gained during the chronic heart failure patient journey, which is particularly characteristic of most heart transplant recipients. The current sample represents a highly selected population, with previous experience in the healthcare system. At Semmelweis University Heart and Vascular Centre (Budapest, Hungary), we implement protocol based patient education sessions before discharging patients, as well as family education session. The multidisciplinary team members (cardiologist, surgeon, anesthesiologist, psychologist, clinical pharmacist, dietetician and heart failure/transplant nurse) regularly educate the patients during the rehabilitation phase as well and they are accessible during the long term follow up. The transplant cardiologist and coordinator team are available 0–24 and they support each heart transplant recipient treated at our facility, in case they are in need of acute medical care. This may be a relevant factor in interpreting our results, which show that participants feel that they can manage their disease well and navigate in the healthcare system easily.

We found lower NVS scores in our sample than the Hungarian representative study, but no significant differences compared to the European representative data (19, 21). The reason for the difference may be due to low educational attainment, because people more likely perform worse on tasks requiring text comprehension and numeracy skills than those with higher educational attainment. The high percentage with lower education level in the sample may be in association with the national healthcare system. Transplantation is covered by health insurance in Hungary, making it more accessible to patients from different socioeconomic backgrounds. Similar results were found in renal transplant population in another study comparing candidate and recipient outcomes with three different HL measurement tools [The Rapid Estimate of Adult Literacy of Medicine-Transplant (REALM-T), NVS test, and the Decision-Making Capacity Assessment Tool (DMCAT)] (25). The authors concluded that there were some discrepancies between these HL tools which may be clinically relevant (25). The NVS is also characterized by being a specific HL tool that measures reading skills, text comprehension and numeracy and this may also be a reason for the overestimation of limited HL (20).

Recently, there has been emerging research on objective and subjective HL, their relationship to each other and how this knowledge can be implemented in clinical practice. A study suggests that subjective and objective, i.e., performance-based HL, are two different concepts and should be treated separately. Objective HL helps patients to recognize low-quality health information and health-related misinformation (26). Another study found that subjective and objective HL and numeracy can also be identified as distinct but related concepts. Its results have also encouraged the use of different types of HL measures on the same patient population to obtain in-depth analysis and a better understanding of HL and health outcomes and their associations (27). Comparison of different HL measurements found low to moderate correlations between different HL screening tools [Test of Functional Health Literacy in Adults (TOFHLA), NVS, HLS-EU-Q47, and Health Literacy Questionnaire (HLQ)], also highlighting the fact that these measure different aspects of health literacy. The researchers suggest that understanding the measurement differences of the different tools can help clinicians to choose the right instrument for their measuring purpose (28).

In addition to the measurement and interpretation of HL, there is growing evidence to support the importance of health behaviors in solid organ transplant (SOT) recipients. Post-transplant smoking is associated with poor outcomes (29). Reported rates of post-transplant smoking range from 1.0 to 73.0% (29). The rate of current smokers in our sample was exceptionally low (2%), which is similar to the findings of the BRIGHT Study (14, 30). This implies that most patients adhere to the strong recommendations of clinicians against smoking, the current ISHLT guideline also supports smoking abstinence (31). While current ISHLT guidelines recommend limiting alcohol consumption to 1–2 drinks per day, a new WHO statement supports total alcohol abstinence for the general population (31, 32). According to a recent study, any amount of alcohol consumption is associated with increased cardiovascular disease and cancer risk (33, 34). On average, 23.6% of SOT recipients consumed alcohol after their transplant, with limited data available regarding the HTx population (35). Our findings are similar, with a quarter of our sample having drunk in the past month. Current evidence on the adverse effect of any amount of alcohol consumed should be presented to HTx recipients, and patient education should also focus on demonstrating the potential negative effects of any amount of alcohol consumed, and guidelines should be updated accordingly.

In SOT recipients, some research suggests that patients maintain low physical activity levels for years after surgery despite the negative health outcomes of low physical activity (36, 37). In our study, 50% of patients performed sufficient physical activity according to the current ISHLT guidelines; however, 50% of patients did not perform sufficient physical activity. The BRIGHT study found similar results (30). Around one fifth of our sample does not do any exercise, which is less than half the prevalence of the Hungarian population (59%) (38). Finding ways to support patients to engage in sufficient physical activity can have huge health benefits by reducing metabolic, hemodynamic, and other risk factors contributing to non-communicable diseases. A study mentions that e-health programs could be a possible aid in achieving these goals (39, 40).

With regard to the relationship between the measured aspects of HL and health behaviors, we found no significant or even marginally significant relationships between the two. This is in line with the findings of HLS EU and the BRIGHT Study in terms of smoking and alcohol consumption (21, 30). The BRIGHT Study, however, found that heart transplant recipients with adequate HL were more likely to be sufficiently active physically (14). In another study, inadequate HL was not found to be significantly associated with health risk behaviors (alcohol, smoking, sedentary lifestyle) and psychosocial factors (peer influence, social norms) were mentioned as possible causes that may influence these behaviors and neutralize the effect of HL (41). Our findings also indicate that in our sample, patient decisions about health behaviors may depend on unexplored psychosocial factors other than health literacy itself.

We found that better subjective HL was associated with better QoL (*p* < 0.05) in the overall index and sub-indices of HLS-EU-Q47. However, objective HL as measured by NVS was not associated with QoL. This may be due to the fact that objective and subjective HL reflect different skills and QoL is also influenced by multiple factors, such as subjective opinion, physical condition, as well as social and economic factors, etc. (42, 43).

Our study focuses on measuring the health literacy of heart transplant recipients and our results and conclusions support the need for establishing future interventions. Improving HL requires a complex assessment process, a multi-faceted approach for both caregivers and stakeholders. As clinicians, it is not only essential to measure HL and consider HL when evaluating a patient but also to be aware of what we measure and how we interpret the results. The analysis of the responses of the HLS-EU-Q47 questions can be useful in identifying the weakest HL domain, characterized by a sub-index of patients with overall excellent or good HL. It may be helpful to design tailored, domain-specific interventions aimed at understanding. However, the NVS results reflect patients with a high potential for misunderstanding or misusing written health information. For patients with lower NVS scores, it may be more effective to combine verbal information with a visual component, rely less on written patient information leaflets, and educate them about the importance of using only reliable health information sources. Our study can be a strong motivating factor for the development of specific HL measurement tools for HTx patients in the future. The use of questionnaire-based measurement tools and the assessment of other psychosocial factors affecting adherence can support a holistic approach that is inevitable in complex care planning.

It would also be important to educate and involve the multidisciplinary post-transplant team (psychologist, clinical pharmacist, dietetician) in evaluating, interpreting and implementing the results of our study and they could also contribute to educating low HL patients about the important health risks, medication regimens.

The primary caregiver also has an important role in post-transplant medical management. The simultaneous health literacy evaluation and interpretation of the primary caregiver could help with further understanding of HL in the transplant population.

In the future, beyond the concept of the Stanford Integrated Psychosocial Assessment for Transplant, large, international, multicenter studies would be needed to measure the predictive value of instruments measuring psychosocial status (44).

There are some limitations to our study. The number and characteristics of individuals who refused participation were not recorded. As a result, we cannot determine whether any selection bias may have occurred during data collection. Because of its cross-sectional design, we could not examine the changes in HL of our sample at different time points after transplant. Therefore, we could not establish causality in the determinants of changing HL either. Another limitation is that we only studied patients from one transplantation center, the gender ratio was not equal, which was counterbalanced by the relatively large sample size. The HL tools used in our study had a more general and less disease-specific structure. There is not any disease-specific HL assessment tool for heart transplant recipients. Comparisons of health literacy with national and international reference data should be interpreted with caution due to potential methodological differences, such as sampling procedures, timing of data collection, and characteristics of the studied populations. Also, there are differences in the degree of education and literacy among countries, this factor should be considered when interpreting our results. Furthermore, comparisons with other studies may be affected by ecological fallacies and uncontrolled confounding factors, which should be considered when interpreting the results. The use of a single-item measure to assess quality of life limits reliability and content validity, and does not allow for the identification of specific domains contributing to the overall rating. The potential impact of social desirability bias was not addressed, which may be considered a limitation given the interviewer-administered data collection. Finally, our sample represents a highly selected group of heart transplant recipients, which limits the generalizability of the findings.

## Data Availability

The raw data supporting the conclusions of this article will be made available by the authors, without undue reservation.

## Footnotes

^1^In order to assess the robustness of Model 2, a binary logistic regression analysis was also conducted. The dependent variable was health literacy measured by the Newest Vital Sign (NVS) test, dichotomized as limited vs. adequate, while the predictors included sex (reference: male), age, education level (reference: primary education), and time since heart transplantation (in months). According to the results, the model proved to be statistically significant (*χ*^2^(4) = 32.38, *p* < 0.001), explained a substantial proportion of the variance (Nagelkerke *R*^2^ = 0.375), and demonstrated acceptable fit based on the Hosmer–Lemeshow test (*χ*^2^(8) = 5.55, *p* = 0.698). The overall classification accuracy was 73.5%, correctly identifying 78.0% of participants with limited health literacy and 68.8% of those with adequate literacy. In this model, both age and education level emerged as significant predictors: each additional year of age was associated with a 13% decrease in the odds of having adequate health literacy (*OR* = 0.87, 95% CI [0.82, 0.93], *p* < 0.001), and participants with at least secondary education had over three times higher odds of adequate health literacy compared to those with only primary education (*OR* = 3.32, 95% CI [1.23, 8.97], *p* = 0.018). Neither sex (*OR* = 1.11, 95% CI [0.30, 4.12], *p* = 0.873) nor time since transplantation (*OR* = 1.01, 95% CI [0.99, 1.02], *p* = 0.399) was significantly associated with the outcome.

## References

[1] World Health Organization. Health promotion glossary of terms 2021. Geneva: World Health Organization (2021). 6 p.

[2] Baccolini V Rosso A Di Paolo C Isonne C Salerno C Migliara G . What is the prevalence of low health literacy in European Union member states? A systematic review and meta-analysis. J Gen Intern Med. (2021) 36:753–61. doi: 10.1007/s11606-020-06407-8, 33403622 PMC7947142

[3] Koltai JA Kun E. A magyarországi egészségértés nemzetközi összehasonlításban. [“Hungarian health literacy in international comparison.”]. Egészségfejlesztés. (2016) 57:3–20. doi: 10.24365/ef.v57i3.62 27936881

[4] Nutbeam D Lloyd JE. Understanding and responding to health literacy as a social determinant of health. Annu Rev Public Health. (2021) 42:159–73. doi: 10.1146/annurev-publhealth-090419-102529, 33035427

[5] Griese L Schaeffer D. Health literacy and chronic disease: a comparison of somatic and mental illness. Front Public Health. (2025) 13:1523723. doi: 10.3389/fpubh.2025.1523723, 40041195 PMC11876042

[6] Larsen MH Mengshoel AM Andersen MH Borge CR Ahlsen B Dahl KG . “A bit of everything”: health literacy interventions in chronic conditions - a systematic review. Patient Educ Couns. (2022) 105:2999–3016. doi: 10.1016/j.pec.2022.05.008, 35641366

[7] Walters R Leslie SJ Polson R Cusack T Gorely T. Establishing the efficacy of interventions to improve health literacy and health behaviours: a systematic review. BMC Public Health. (2020) 20:1040. doi: 10.1186/s12889-020-08991-0 32605608 PMC7329558

[8] Chisholm-Burns MA Spivey CA Pickett LR. Health literacy in solid-organ transplantation: a model to improve understanding. Patient Prefer Adherence. (2018) 12:2325–38. doi: 10.2147/PPA.S183092, 30464420 PMC6229143

[9] Cocchieri A Cristofori E Nurchis MC Nursing And Public Health Group Damiani G Cesare M. Nursing complexity and health literacy as determinants of patient outcomes: a prospective one-year multicenter cohort study. Nurs Rep. (2025) 15:135. doi: 10.3390/nursrep15040135, 40333082 PMC12029856

[10] Lorenz EC Petterson TM Schinstock CA Johnson BK Kukla A Kremers WK . The relationship between health literacy and outcomes before and after kidney transplantation. Transplant Direct. (2022) 8:e1377. doi: 10.1097/TXD.0000000000001377, 36204189 PMC9529030

[11] Bittermann T Dwinnells K Chadha S Wolf MS Olthoff KM Serper M. Low health literacy is associated with frailty and reduced likelihood of liver transplant listing: a prospective cohort study. Liver Transpl. (2020) 26:1409–21. doi: 10.1002/lt.25830, 32567232 PMC8809114

[12] Serper M Patzer RE Reese PP Przytula K Koval R Ladner DP . Medication misuse, nonadherence, and clinical outcomes among liver transplant recipients. Liver Transpl. (2015) 21:22–8. doi: 10.1002/lt.24023, 25312406 PMC5831120

[13] Demian MN Shapiro RJ Thornton WL. An observational study of health literacy and medication adherence in adult kidney transplant recipients. Clin Kidney J. (2016) 9:858–65. doi: 10.1093/ckj/sfw076, 27994867 PMC5162408

[14] Cajita MI Denhaerynck K Dobbels F Berben L Russell CL Davidson PM . Health literacy in heart transplantation: prevalence, correlates and associations with health behaviors-findings from the international BRIGHT study. J Heart Lung Transplant. (2017) 36:272–9. doi: 10.1016/j.healun.2016.08.024 27773449

[15] Lennerling A Kisch AM Forsberg A. Health literacy among Swedish lung transplant recipients 1 to 5 years after transplantation. Prog Transplant. (2018) 28:338–42. doi: 10.1177/1526924818800043, 30205755

[16] McIlvennan CK. Looking on the BRIGHT side of health literacy in patients with cardiac transplantation: Where are we and where do we need to go? J Heart Lung Transplant. (2017) 36:253–5. doi: 10.1016/j.healun.2016.10.002 27876414

[17] Sørensen K Pelikan JM Röthlin F Ganahl K Slonska Z Doyle G . Health literacy in Europe: comparative results of the European health literacy survey (HLS-EU). Eur J Pub Health. (2015) 25:1053–8. doi: 10.1093/eurpub/ckv043, 25843827 PMC4668324

[18] Sørensen K Van den Broucke S Pelikan JM Fullam J Doyle G Slonska Z . Measuring health literacy in populations: illuminating the design and development process of the European health literacy survey questionnaire (HLS-EU-Q). BMC Public Health. (2013) 13:948. doi: 10.1186/1471-2458-13-948, 24112855 PMC4016258

[19] Koltai JA Kun E. Az egészségértés gyakorlati mérése Magyarországon és nemzetközi összehasonlításban. [The practical measurement of health literacy in Hungary and in international comparison]. Orv Hetil. (2016) 157:2002–6. doi: 10.1556/650.2016.30563 27936881

[20] Weiss BD Mays MZ Martz W Castro KM DeWalt DA Pignone MP . Quick assessment of literacy in primary care: the newest vital sign. Ann Fam Med. (2005) 3:514–22. doi: 10.1370/afm.405 16338915 PMC1466931

[21] HLS-EU CONSORTIUM (2012): Comparative report of health literacy in eight EU member states. The European Health Literacy Survey HLS-EU. Available online at: http://cpme.dyndns.org:591/adopted/2015/Comparative_report_on_health_literacy_in_eight_EU_member_states.pdf (Accessed August 14, 2024).

[22] Koo TK Li MY. A guideline of selecting and reporting intraclass correlation coefficients for reliability research. J Chiropr Med. (2016) 15:155–63. doi: 10.1016/j.jcm.2016.02.012, 27330520 PMC4913118

[23] Faul F Erdfelder E Buchner A Lang AG. Statistical power analyses using G*power 3.1: tests for correlation and regression analyses. Behav Res Methods. (2009) 41:1149–60. doi: 10.3758/BRM.41.4.1149, 19897823

[24] Cohen J. A power primer. Psychol Bull. (1992) 112:155–9. doi: 10.1037/0033-2909.112.1.155, 19565683

[25] Kazley AS Hund JJ Simpson KN Chavin K Baliga P. Health literacy and kidney transplant outcomes. Prog Transplant. (2015) 25:85–90. doi: 10.7182/pit2015463, 25758806

[26] Schulz PJ Pessina A Hartung U Petrocchi S. Effects of objective and subjective health literacy on patients' accurate judgment of health information and decision-making ability: survey study. J Med Internet Res. (2021) 23:e20457. doi: 10.2196/20457, 33475519 PMC7861996

[27] Waters EA Biddle C Kaphingst KA Schofield E Kiviniemi MT Orom H . Examining the interrelations among objective and subjective health literacy and numeracy and their associations with health knowledge. J Gen Intern Med. (2018) 33:1945–53. doi: 10.1007/s11606-018-4624-2, 30120636 PMC6206359

[28] Jessup RL Beauchamp A Osborne RH Hawkins M Buchbinder R. Health literacy measurement: a comparison of four widely used health literacy instruments (TOFHLA, NVS, HLS-EU and HLQ) and implications for practice. Aust J Prim Health. (2024) 30:PY22280. doi: 10.1071/PY22280 39699997

[29] Duerinckx N Burkhalter H Engberg SJ Kirsch M Klem M-L Sereika SM . Correlates and outcomes of posttransplant smoking in solid organ transplant recipients: a systematic literature review and meta-analysis. Transplantation. (2016) 100:2252–63. doi: 10.1097/TP.0000000000001335, 27479162

[30] Helmy R Duerinckx N De Geest S Denhaerynck K Berben L Russell CL . The international prevalence and variability of nonadherence to the nonpharmacologic treatment regimen after heart transplantation: findings from the cross-sectional BRIGHT study. Clin Transpl. (2018) 32:e13280. doi: 10.1111/ctr.13280, 29754400

[31] Velleca A Shullo MA Dhital K Azeka E Colvin M DePasquale E . The International Society for Heart and Lung Transplantation (ISHLT) guidelines for the care of heart transplant recipients. J Heart Lung Transplant. (2023) 42:e1–e141. doi: 10.1016/j.healun.2022.10.015, 37080658

[32] Anderson BO Berdzuli N Ilbawi A Kestel D Kluge HP Krech R . Health and cancer risks associated with low levels of alcohol consumption. Lancet Public Health. (2023) 8:e6–7. doi: 10.1016/S2468-2667(22)00317-6, 36603913 PMC9831798

[33] Rovira P Rehm J. Estimation of cancers caused by light to moderate alcohol consumption in the European Union. Eur J Pub Health. (2021) 31:591–6. doi: 10.1093/eurpub/ckaa236, 33338220

[34] Biddinger KJ Emdin CA Haas ME Wang M Hindy G Ellinor PT . Association of habitual alcohol intake with risk of cardiovascular disease. JAMA Netw Open. (2022) 5:e223849. doi: 10.1001/jamanetworkopen.2022.3849 35333364 PMC8956974

[35] Dobbels F Denhaerynck K Klem ML Sereika SM de Geest S de Simone P . Correlates and outcomes of alcohol use after single solid organ transplantation: a systematic review and meta-analysis. Transplant Rev (Orlando). (2019) 33:17–28. doi: 10.1016/j.trre.2018.09.003 30472153

[36] Gustaw T Schoo E Barbalinardo C Rodrigues N Zameni Y Motta VN . Physical activity in solid organ transplant recipients: participation, predictors, barriers, and facilitators. Clin Transpl. (2017) 31:e12929. doi: 10.1111/ctr.12929, 28185297

[37] Berben L Engberg SJ Rossmeissl A Gordon EJ Kugler C Schmidt-Trucksäss A . Correlates and outcomes of low physical activity posttransplant: a systematic review and Meta-analysis. Transplantation. (2019) 103:679–88. doi: 10.1097/TP.0000000000002543, 30461720

[38] European Commission. Special Eurobarometer 525 sport and physical activity European Commission, directorate-general for education, youth, sport and culture. (2022). Available online at: https://europa.eu/eurobarometer/api/deliverable/download/file?deliverableId=83654 (Accessed: November 11, 2024).

[39] Naci H Ioannidis JP. Comparative effectiveness of exercise and drug interventions on mortality outcomes: metaepidemiological study. Br J Sports Med. (2015) 49:1414–22. doi: 10.1136/bjsports-2015-f5577rep, 26476429 PMC4680125

[40] Makai A Füge K Breitenbach Z Betlehem J Ács P Lampek K . The effect of a community-based e-health program to promote the role of physical activity among healthy adults in Hungary. BMC Public Health. (2020) 20:1059. doi: 10.1186/s12889-020-08750-1, 32799879 PMC7429904

[41] Wolf MS Gazmararian JA Baker DW. Health literacy and health risk behaviors among older adults. Am J Prev Med. (2007) 32:19–24. doi: 10.1016/j.amepre.2006.08.024, 17184964

[42] Rosenberger EM Fox KR DiMartini AF Dew MA. Psychosocial factors and quality-of-life after heart transplantation and mechanical circulatory support. Curr Opin Organ Transplant. (2012) 17:558–63. doi: 10.1097/MOT.0b013e3283564f45, 22890039 PMC3612288

[43] Ross H Abbey S De Luca E Mauthner O McKeever P Shildrick M . What they say versus what we see: "hidden" distress and impaired quality of life in heart transplant recipients. J Heart Lung Transplant. (2010) 29:1142–9. doi: 10.1016/j.healun.2010.05.009, 20580266

[44] Maldonado JR Sher Y Lolak S Swendsen H Skibola D Neri E . The Stanford integrated psychosocial assessment for transplantation: a prospective study of medical and psychosocial outcomes. Psychosom Med. (2015) 77:1018–30. doi: 10.1097/PSY.0000000000000241, 26517474

